# User-Driven Relay Beamforming for mmWave Massive Analog-Relay MIMO

**DOI:** 10.3390/s23021034

**Published:** 2023-01-16

**Authors:** Masashi Iwabuchi, Yoghitha Ramamoorthi, Kei Sakaguchi

**Affiliations:** 1 NTT Access Network Service Systems Laboratories, Nippon Telegraph and Telephone Corporation, Yokosuka 239-0847, Japan; 2 Tokyo Institute of Technology, Tokyo 152-8552, Japan

**Keywords:** 6G, massive analog-relay MIMO, amplifier-and-forward, cooperative awareness message, location-based beamforming, beam tracking

## Abstract

Sixth-generation mobile communication (6G) aims to further improve capacity and reliability by controlling the radio propagation environment. Millimeter wave (mmWave) high-frequency band communication offers large bandwidth at the cost of high attenuation, even for smaller distances. Due to this, fewer multiple input multiple outputs (MIMO) multiplexing is possible at the base station (BS). Distributed analog relay nodes with beamforming capability improve the received power and MIMO multiplexing of mmWave communication. Due to limited signal processing, the analog relay node cannot perform beam search and tracking using these mmWave reference signals. The beam search and tracking are possible at BS or user equipment at the cost of increased control overhead. To reduce this overhead and provide relay-based 6G communication, we propose user-driven relay beamforming methods which can obtain the benefits of a massive analog relay MIMO. Assuming vehicular-to-everything (V2X) as a 6G application, we considered a relay-beam control method that uses the user information (location, velocity, acceleration, and direction of the terminal) contained in intelligent transport systems (ITS) messages called Cooperative Awareness Message (CAM). Simulation results show that the proposed method significantly reduces the overhead and the obtains benefits of the massive analog-relay MIMO. Furthermore, the accuracy of CAM’s location information, the control period, and the effects of UE mobility are evaluated and presented. The results also show that the proposed method can work effectively in future V2X applications.

## 1. Introduction

Research and development on the sixth-generation mobile communication system (6G) have started in some research communities [1,2,3]. Further technological evolution related to millimeter-wave (mmWave) communications is required for the 6G era. In the fifth-generation mobile communication system (5G), one fundamental approach for enhancing the data rate and the channel capacity is to utilize higher-frequency bands such as the 28 GHz bands. High-capacity communication using the mmWave band is required for vehicular-to-everything (V2X) applications such as autonomous driving and remote driving that generate large amounts of data communication [4]. However, 5G is the first generation that applies mmWave bands to radio access networks. Therefore, some issues remain regarding the use of these bands. The typical examples are smaller coverage areas caused by significant pathloss and multiple-input–multiple-output (MIMO) stream reduction due to less scattering [5].

As a technical field for enhancing the potential of communication systems, the concept of controllable radio environments is attracting attention [6,7]. This concept assumes a large number of relay devices to control the radio environment. There are several technologies for wireless relay. The Decode-and-Forward (DF) relay method is one of the typical methods, and it has been discussed as integrated access and backhaul (IAB) in the standardization of the 3rd Generation Partnership Project (3GPP) Release 16 [8]. The DF relay can reduce noise and flexibly control radio resources through its decoding and re-encoding operations. However, this operation causes delays due to signal processing on relay nodes (RNs). Furthermore, since the cost is higher than other relay methods, it is not suitable for deploying a large number of RNs. Reconfigurable Intelligent Surface (RIS), a reflector that can switch the reflection direction of radio waves using meta-surface technologies, is also a remarkable solution [9,10] for improving signal quality. RIS extends coverage by forming extremely sharp beams instead of amplifying signal power. This requirement limits RIS usage in high-mobility scenarios such as V2X. In order to address this issue in high-mobility scenarios, we focus on the Amplifier-and-Forward (AF) relay method. These AF relays are easy to implement, and the latest development of AF relays were discussed in 3GPP Release 18 [11]. It is termed a smart repeater that consists of a repeater with beamforming abilities. Radio environments can be controlled by spatially distributing RNs and operating them dynamically [12]. In [13], the enhancement of single-user MIMO utilizing a massive AF relay with beamforming called massive analog-relay MIMO is proposed. However, a control method in a system with multiple analog relays has not been considered in these studies.

This paper proposes a user-driven relay beamforming method for the massive analog-relay MIMO to improve mmWave communications for V2X applications. To control relay beamforming, a network-driven approach is considered. This approach manages the relay beams from the network side. However, since the BSs and the RNs are remotely distributed, the control latency and overhead increase proportionately to the beam candidates and the number of RNs. To efficiently control multiple RNs, we propose the user-driven relay beamforming method. Assuming V2X applications, the proposed method employs side information, such as location information obtained from Cooperative Awareness Message (CAM) [14], for controlling the relay beam. The CAM is a kind of Intelligent Transport Systems (ITS) message, and the user equipment (UE) can broadcast this message. For V2X applications, the concept of beam alignment between BS and vehicles using location information was discussed in [15,16]. In [15], the location-based approach can significantly reduce the mmWave beam alignment overhead while having marginal performance degradation. However, these studies mainly focus on the initial beam search of the base station (BS) and do not cover the beam operation of the analog relay. Also, there are no specific proposals and evaluations on beam tracking at the RNs. In this paper, we propose a user-driven beam searching and tracking method for massive RNs with low overhead that obtain the benefits of massive relay MIMO.

The rest of this paper is organized as follows. Section 2 describes and formulates the massive analog-relay MIMO. Section 3 proposes the user-driven relay-beam control method for massive RNs. Simulation evaluations are presented and discussed in Section 4. Finally, Section 5 concludes this paper with the possible future works.

## 2. Massive Analog-Relay MIMO

**Figure 1:**
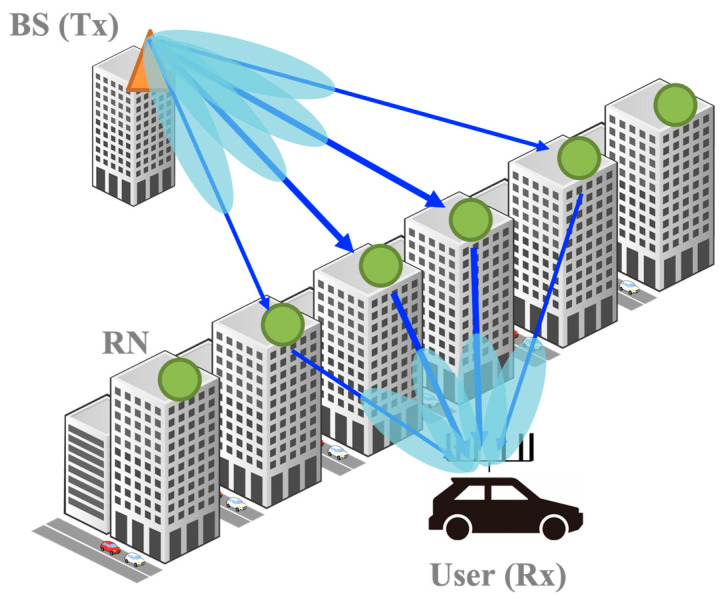
System model of the massive analog-relay MIMO in V2X applications.

**Figure 2:**
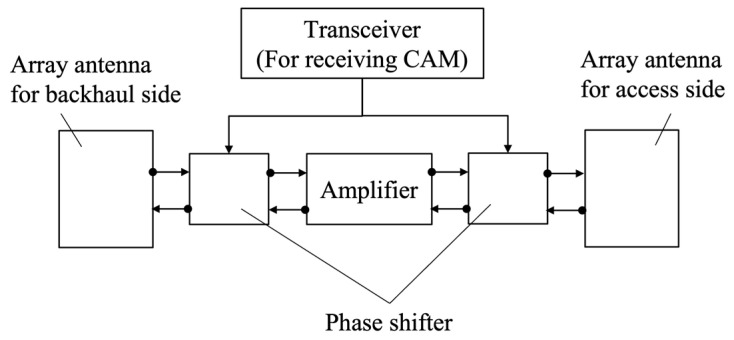
Architecture of the analog RN in massive analog-relay MIMO.

This section describes the massive analog-relay MIMO and formulates the newly generated channel by multiple RNs. Figure 1 shows the system configuration discussed in this paper. The configuration is composed of one BS with *M_t_* antennas, *K* RNs, and one UE with *M_r_* antennas. Full-array hybrid beamforming [17] is assumed in both BS and UE. Figure 2 illustrates the architecture of the analog RNs, such as a radio repeater, assumed in this study. Unlike the DF relay, the AF relay does not involve digital signal processing for mmWave signals and thus, radio signals can be relayed with low latency. To compensate for attenuation due to mmWave, massive analog-relay MIMO performs beamforming in analog RNs. Furthermore, since hardware cost is lower, it is possible to reduce the network cost when considering the distribution of massive RNs. Unlike the conventional AF relay, massive analog-relay MIMO also performs beamforming in analog RNs to compensate for the attenuation of mmWave. The RNs support analog beamforming only. In the analog beamforming, the same analog signal is fed to each antenna, and then the analog phase shifter to steer the signal. In the proposed method described in the next section, the analog RN consists of a transceiver that acquires information to control its analog beamforming. The transceiver is used to receive the CAM and control the relay beam. Since the transceiver is assumed to utilize low-frequency bands used by ITS, it is relatively inexpensive when compared to the mmWave band transceiver.

The signal model of massive analog-relay MIMO is formulated. The weight matrices in the analog domain are $V_{a}\in {\mathbb{C}}^{M_{t}\times N_{s}}$ for BS and $W_{a}\in {\mathbb{C}}^{M_{r}\times N_{s}}$ for UE. Then, *N_s_* represents the number of streams. The weight matrices in digital domain are $V_{d}\in {\mathbb{C}}^{N_{s}\times N_{s}}$ and $W_{d}\in {\mathbb{C}}^{N_{s}\times N_{s}}$ for BS and UE, respectively. The channel between BS and UE is defined as $H\in {\mathbb{C}}^{M_{r}\times M_{t}}$. Assuming downlink (DL) transmission through this paper, the received signal $y\in {\mathbb{C}}^{N_{s}\times 1}$ is represented as follows.

(1) $$y=W_{d}^{H}W_{a}^{H}\left(HV_{a}V_{d}x+n\right)\;=W_{d}^{H}W_{a}^{H}HV_{a}V_{d}x+W_{d}^{H}W_{a}^{H}n\;=W_{d}^{H}\tilde{H}V_{d}x+W_{d}^{H}\tilde{n}$$

where $x\in {\mathbb{C}}^{N_{s}\times 1}$ and $n\in {\mathbb{C}}^{N_{s}\times 1}$ indicates the transmitted signal and noise, respectively.

Next, channel representation in massive analog-relay MIMO is described. Transmitted and received analog beamforming weights of *K* RNs are represented as $U_{r}=\mathrm{diag}\left\{u_{r,k}\right\}\in {\mathbb{C}}^{KL_{r}\times K}$ and $U_{t}=\mathrm{diag}\left\{u_{t,k}\right\}\in {\mathbb{C}}^{KL_{t}\times K}$, respectively. Then, the vector $u_{t,k}\in {\mathbb{C}}^{K_{t}\times 1}$ and $u_{r,k}\in {\mathbb{C}}^{K_{r}\times 1}$ denote analog weights of the *k*-th RN. Using the amplifier gain of the *k*-th RN *g*_k_, the amplifier-gain matrix is defined as $G=\mathrm{diag}\left\{\sqrt{g_{k}}\right\}\in {\mathbb{C}}^{K\times K}$. Given a channel matrix between the BS and the RNs as $H_{1}\in {\mathbb{C}}^{KL_{r}\times M_{t}}$, a channel matrix between the RNs and the UE as $H_{2}\in {\mathbb{C}}^{M_{r}\times KL_{t}}$ and a channel matrix between the BS and the UE as $H_{0}\in {\mathbb{C}}^{M_{r}\times M_{t}}$, the channel matrix in the massive analog-relay MIMO system **H** is represented as follows.

(2) $$H=H_{0}+H_{2}U_{t}GU_{r}^{H}H_{1}$$

Although the analog RN can improve received power, it causes additional noise. Here, if $n_{R}\in {\mathbb{C}}^{K\times 1}$ denotes noise added in the analog RNs, the noise vector **n** in Equation (1) is rewritten as follows.

(3) $$n=H_{2}U_{t}Gn_{R}+n\prime$$

Then, **n**’ is noise at the UE. From above equations, the rate of massive analog-relay MIMO can be expressed as Equation (3). Here, it assumes SVD (singular value decomposition) -MIMO and equal power allocation to each stream.

(4) $$C=\log_{2}\det\left(I+\frac{{\left|W_{d}^{H}\tilde{H}V_{d}\right|}^{2}{\left|x\right|}^{2}}{{\left|W_{d}^{H}\tilde{n}\right|}^{2}}\right)\;=\overset{N_{s}}{\underset{m=1}{\sum }}\log_{2}\left(1+\frac{\lambda_{m}P_{x}}{N_{s}P_{n}}\right)$$

Then, *P_x_* and *P_n_* are transmission power and noise power, respectively. *λ_m_* means *m*-th singular value.

## 3. User-Driven Analog-Relay Beamforming

Massive analog-relay MIMO can improve the channel characteristic by adjusting **U***_t_*, **U***_r_*, and **G** in Equations (2) and (3). This paper mainly focuses on the relay-beamforming matrix **U***_t_* that needs to be dynamically controlled as the UE moves. To track the UE, the RNs control analog beamforming. However, they have no signal-processing function supporting mmWave bands. Therefore, how to properly search the analog beam in the RNs is one of the important issues. One of the possible methods is an exhaustive search, where BS and UE cooperate to measure the channel state or quality for all relay-beam candidates. After searching all channel qualities, the BS selects relay beams and notifies the RNs of the beam information. A problem with this method is increased overhead in proportion to the number of RNs due to the channel measurement and the notification. Since the massive analog-relay MIMO utilizes multiple RNs, the overhead is large. Therefore, such exploratory methods are not suitable.

To solve this issue, this paper proposes a user-driven relay-beamforming method. The proposed approach consists of two phases. Analog-beamforming weights of the RN called relay-beamforming weights are searched in the first phase. To decide the analog-beamforming weights while keeping few overheads, the proposed method utilizes location information. After finding the relay beamforming weights, beamforming weights of the BS and the UE are searched in the second phase. In this phase, the beamforming weights are calculated based on a reference signal (RS), such as channel state information (CSI)-RS. The second phase can occur any number of times before updating the relay-beamforming weights.

There are some limitations of the proposed method. One primary concern is the error of location estimation. It was difficult to apply such an approach because the position estimation error is large in a typical positioning method such as the conventional global positioning system (GPS) and cellular positioning. However, recent positioning technologies discussed in some standards such as 3GPP release 16 [18] and IEEE 802.11az [19], have centimeter-level accuracy. Additionally, V2X applications such as autonomous driving cars require high-precision positioning. Therefore, V2X UE has a function that enables high-precision positioning such as the global navigation satellite system (GNSS) and light detection and ranging (LiDAR) [20]. Considering the recent location estimation techniques in V2X applications, the concern about location estimation error can be solved. Another concern is the inability to support users in non-line-of-sight (NLOS) from RNs. As described later, the proposed method geometrically selects relay beams based on the estimated locations. In NLOS environments, since it is likely that the direct waves from the RN will no longer be dominant, the proposed method may be not suitable to select beams for users in NLOS. However, we assume that the proposed method will be applied to mmWave communications. The performance of mmWave communication is greatly degraded in NLOS environments, and it is basically applied to line-of-sight (LOS) communications. Also, the experimental results shown in [5] show that analog beams often follow the user’s position. In this paper, we aim to reduce the overhead of controlling analog beams at relay nodes. The proposed method is suitable to achieve the purpose.

### 3.1. User-Driven Relay-Beam Selection

**Figure 3:**
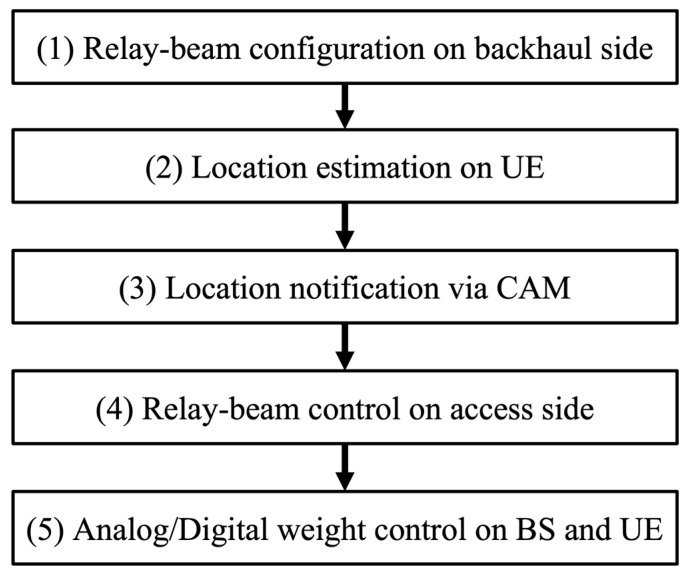
Control flow of the user-driven relay-beam selection.

The user-driven relay-beam selection method involves two phases. The first phase consists of four steps to compute the beamforming weight of RNs. The second phase consists of one step that determines the beamforming weights of BS and UE. Figure 3 shows the control flow of the proposed method. The overall flow consists of five steps as follows.

1. Relay-beam configuration on backhaul side

Assuming a static radio environment where the BS and the RNs are fixed, the same relay beam continues to be selected on the backhaul side. Therefore, it is sufficient to adjust the beam when installing the RN or after a long period. Even if the RN moves, the beam on the backhaul side can be determined in the same way as in Step 4 (it is necessary to replace the UE in Step 4 with BS.). Then, the RN needs the location-estimation function to know the location of the BS that should connect to it. The assumption of the fixed RN is reasonable, since the RN rarely moves in actual deployment. Thus, the beam on the backhaul side is determined appropriately in advance.

2. UE location estimation

The relay beam on the access side needs to track the target UE. The proposed method controls the relay beam by utilizing location information. In this paper, utilizing high-precision positioning such as LiDAR is assumed on the UE. Note that it is assumed the location information of the BS and each RN are obtained when equipment is installed. The estimated location $p_{m}=\left(x_{m},\;y_{m}\right)$ includes the estimation error $p_{e}$ and is presented as follow.

(5) $$p_{m}=p+p_{e}\;$$

Then, p is a true location of the UE.

3. Notification by CAM

The location information estimated in step 3 needs to be notified to the RNs. To reduce the control overhead, we propose notification utilizing CAM in V2X. The CAM includes vehicle location, speed, acceleration, and direction. The CAM is transmitted from the UE via NR-V2X [21,22]. The typical message size of the CAM is from 350 to 400 bytes. The transmission cycle of the CAM is from 100 to 1000 ms.

4. Relay-beam configuration on access side

After receiving location information from the UE, the RNs calculates the direction of the analog beamforming that should be aimed at the UE. The direction is geometrically determined by using the UE and the RN location information according to the following formulas. For simplicity, a two-dimensional plane is assumed, and only the azimuth angle is described.

(6) $$\varphi =\arctan\left(\frac{y_{1}-y_{0}}{x_{1}-x_{0}}\right)-\varphi_{0}$$

(7) $$i\in \underset{0\leq n\leq N,\;n\in \mathbb{N}\;}{\arg\;\min}\left(\left|{\tilde{\varphi }}_{n}-\varphi_{0}\right|\right)$$

where the coordinates (*x*_0_, *y*_0_) and (*x*_1_, *y*_1_) are RN’s and UE’s locations, respectively. An angle $\varphi_{0}$ is UE-side antenna boresight of the RN. The RN knows (*x*_0_, *y*_0_) and $\varphi_{0}$ in advance. Based on φ calculated in Equation (6), the beam number *i* with the closest beam angle to φ is selected. In Equation (7), *N* is the number of UE-side beam candidates on the RN.

Steps 1–4 described above belong to the first phase of the proposed method which computes the beamforming weight of the RNs. Through the first phase, new coverage and paths are formed between the BS and the UE via the RNs. The final step computing beamforming weights of BS and UE in the second phase is explained below.

5. Analog and digital weight control on the BS and the UE

The final step, which corresponds to the second phase of the proposed method, is processing the BS and the UE. Since the processing can be applied independently from the relay-beam selection, the processing can be performed based on a procedure specified in the standard such as 5G NR [23]. However, since massive analog-relay MIMO utilizes multiple beams, additional consideration is required for beam selection at the BS and the UE. To select multiple beams to maximize capacity, a sequential quasi-optimization procedure as in [13] is considered.

### 3.2. Relay-Beam Tracking

**Figure 4:**
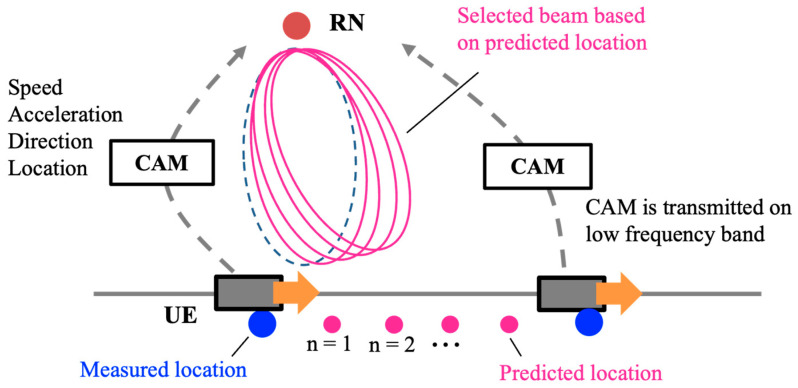
Relay-beam tracking utilizing UE location, speed, acceleration, and direction contained in CAM.

Beam tracking is also an important issue in analog relay beamforming. In many cases, the BS tracks the beam using RNs after the beam search. However, the analog RN cannot utilize RS because it has no signal processing for millimeter-wave bands. As mentioned above, since the transmission cycle of the CAM is relatively long, only the proposed beam search causes the location error due to the UE mobility and reduces the gain from massive RNs. Therefore, a relay-beam tracking method, that utilizes the UE information contained in the CAM is proposed. Figure 4 shows the proposed tracking method. The RN can acquire the UE’s location $p_{m}\left(t_{0}\right)$, speed $v\left(t_{0}\right)$, acceleration $a\left(t_{0}\right)$ and direction $\hat{d}\left(t_{0}\right)$ from the received CAM. Here, *t*_0_ means the time when these values are measured. From this information, it is possible to predict the location of the UE. The predicted location $\tilde{p}\left(t\right)$ can be calculated from Equation (8).

(8) $$\tilde{p}\left(t_{0}+nt_{p}\right)=p_{m}\left(t_{0}\right)+nt_{p}\left(v\left(t_{0}\right)+\frac{1}{2}a\left(t_{0}\right)nt_{p}\right)\hat{d}\left(t_{0}\right),\;n\in \mathbb{Z},\;n\geq 0\;$$

where *t_p_* is the prediction cycle. The UE’s direction from the RN can be estimated from Equations (6) and (8), and the relay beam can be updated as shown below.

(9) $$\varphi =\arctan\left(\frac{{\tilde{p}}_{y}\left(t+nt_{p}\right)-y_{0}}{{\tilde{p}}_{x}\left(t+nt_{p}\right)-x_{0}}\right)-\varphi_{0}\;$$

### 3.3. Overhead Analysis

The purpose of the proposed method is to reduce overhead in massive analog-relay MIMO. To focus on the relay-beam search in the step (4) described in the previous section, analog-beam search on the BS and the UE is ignored. It assumes they know the optimal beam for each RN. As a conventional method, we introduce a network-driven exhaustive search. Here, it is assumed that the beamforming settings on the RN are ideally performed using the control signal from the BS. Assuming a sequential quasi-optimization procedure, the cumulative overhead time *T_o_* is expressed as follows.

(10) $$T_{o}=T_{rs}L_{rn}\overset{N_{s}}{\underset{n=1}{\sum }}\left(K-n+1\right)\;$$

where *T_rs_* is a length of the reference signal for beam search, and *N_s_* is the number of streams. *L_rn_* is the number of beam candidates on the RN. The above formula expresses the overhead when the reference signals are continuously transmitted. In practice, the reference signal is transmitted intermittently, and so the control time takes longer. Next, the proposed user-driven beam search of relay nodes is discussed. Since the UE only broadcasts its self-estimated location information, the overhead time is given by the following equation.

(11) $$T_{o}=T_{msg}$$

Then, *T_msg_* is length of a message including location information sent by the UE. From Equation (11), the proposed method has constant overhead regardless of the number of RNs around the UE, and it is an effective method for massive analog-relay MIMO that utilizes many RNs. In addition, all the overhead of the conventional method always occurs in mmWave bands where data communication is performed, while the overhead of the proposed method can alternatively be consumed in different frequency bands.

## 4. Simulation

### 4.1. Simulation Configuration

**Figure 5:**
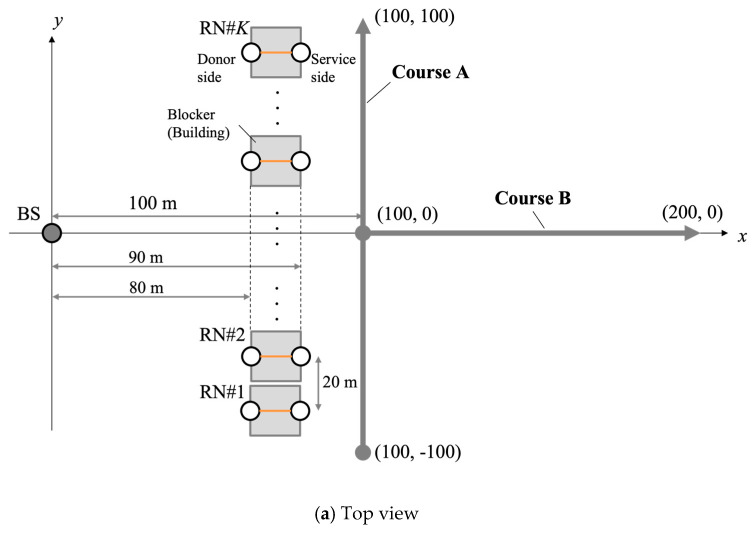

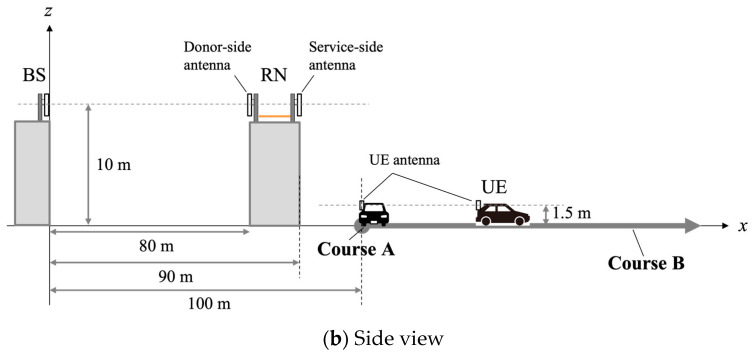
Simulation model.

The proposed method was evaluated by computer simulation using MATLAB^®^ R2021a. Figure 5 shows the simulation model. BS and UE are deployed as shown in Figure 5. We consider a single UE in this simulation. However, the proposed method can also be evaluated for multiple users sequentially. We consider 12 RNs placed between the BS and the UE. The RNs are placed at equal intervals on a straight line as shown in Figure 5. There is always LOS between the BS and the RNs, and between the RNs and the the UE. On the other hand, there is always NLOS between the BS and the UE. It is assumed that the antenna boresight on the BS and the RNs (UE side) are “+x” direction, and the antenna boresight on the RNs (BS side) and the UE are “−x” direction. Elevation angle of antenna tilt is set to zero in all nodes. Table 1 lists the simulation parameters. For the analog beam direction, rough beamforming with 15-degree increments and fine beamforming with 1-degree increments were evaluated. Rough beamforming is often used for initial access, but it is a factor of characteristic deterioration because the beam direction is not properly directed to the UE. In recent years, a phased array antenna module that can adjust the beam direction in 1-degree increments was also developed [24], so we basically considered fine beamforming in the evaluations. The antenna polarization was single polarization. SVD-MIMO is applied as digital processing technique at the BS and the UE. We assume that the CSI can be ideally estimated.

### 4.2. Massive Analog-Relay MIMO with the Proposed Method

**Figure 6:**
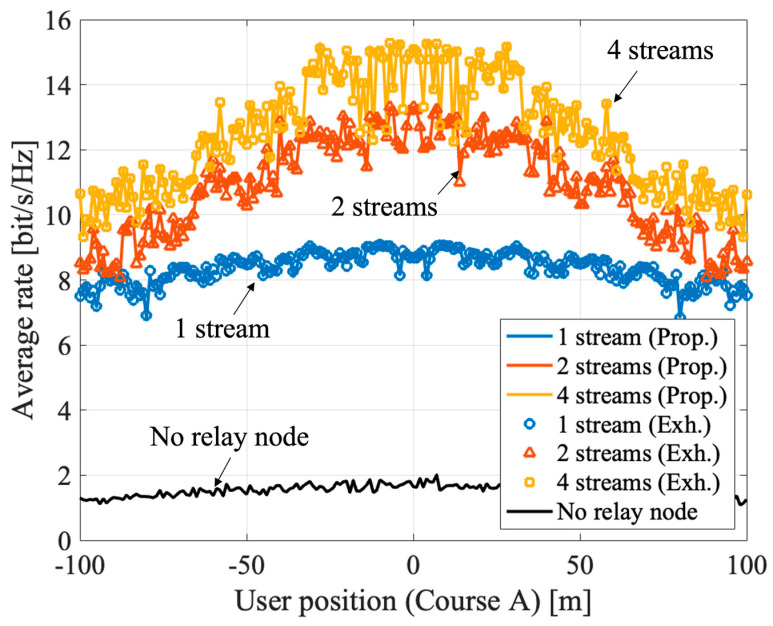
Average rate of massive relay MIMO with the proposed method in course A.

**Figure 7:**
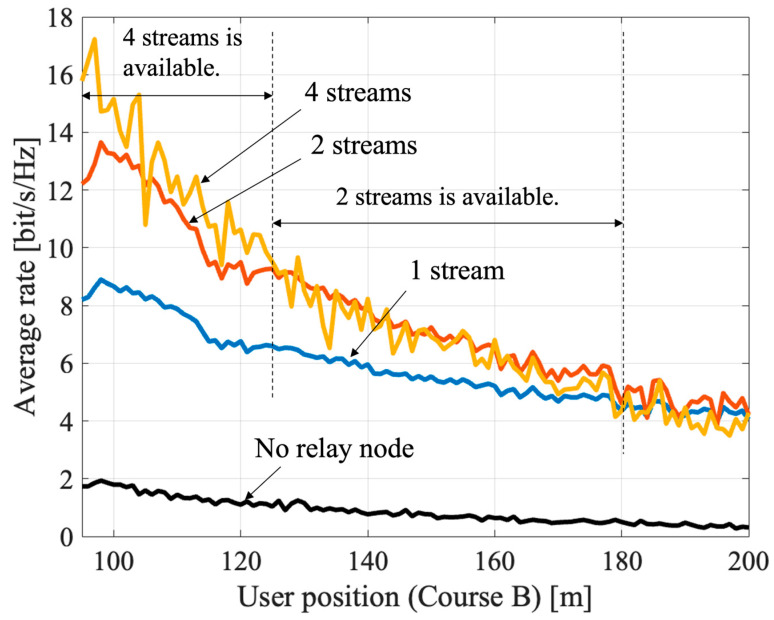
Average rate of massive relay MIMO with the proposed method in the course B.

Figure 6 and Figure 7 show the results of massive analog-relay MIMO with the proposed method. The results show that the proposed method can improve the rate in the massive analog-relay MIMO. Here, it is assumed that all RNs receive ideal location information from the UE. Also, the number of streams is fixed without rank adaptation, and the power is equally distributed to each stream. The average rate is calculated based on Equation (4), and the average value of 100 samples is plotted at each point.

Figure 6 shows the UE coordinates x=100 m, −100 m≤y≤100 m. The average rate is improved in the range where the RNs are installed (from −70 m to +70 m). The RNs extend coverage and improve overall performance. Comparing the 1-stream and the 4-stream transmission near the coordinates (100, 0) of the UE, we can see that the improvement is about 72% compared to the 1-stream case. The reason is improvement of spatial multiplexing by passing through multiple RNs. When y = −100 m or +100 m, the benefits of spatial multiplexing are hardly obtained because the directions of arrival of each route that passes through each RN are close to each other. It can be solved by distributing more RNs. Figure 7 shows the characteristics when the terminal position is 100 m≤x≤200 m, y=0 m. As in Figure 7, the shorter the distance between the terminal and the RN, better the capacity. If V2X are assumed, the RN is expected to be installed in a roadside unit, so the distance between the relay node and the UE is about 5–20 m. Therefore, the benefits of massive relay MIMO can be fully obtained in V2X use cases. The proposed method can be applicable to multi-user scenarios also. In the massive analog-relay MIMO, the distance between RNs and users are one of the primary factors to define the performance of the system. Another important parameter to consider while analyzing performance is the distance between the users. If the users are in proximity, there is a greater probability that the signals pass through common or neighboring RNs. Therefore, the link between the RNs and the users has higher interference. However, if the users are far apart, the interference between users is negligible because each user would be associated with a different RN cluster, and thereby multi-user transmission is possible. Thus, in this case, spatial multiplexing gain can be obtained by the proposed method even when multi-user MIMO transmission is performed in the massive analog-relay MIMO.

**Figure 8:**
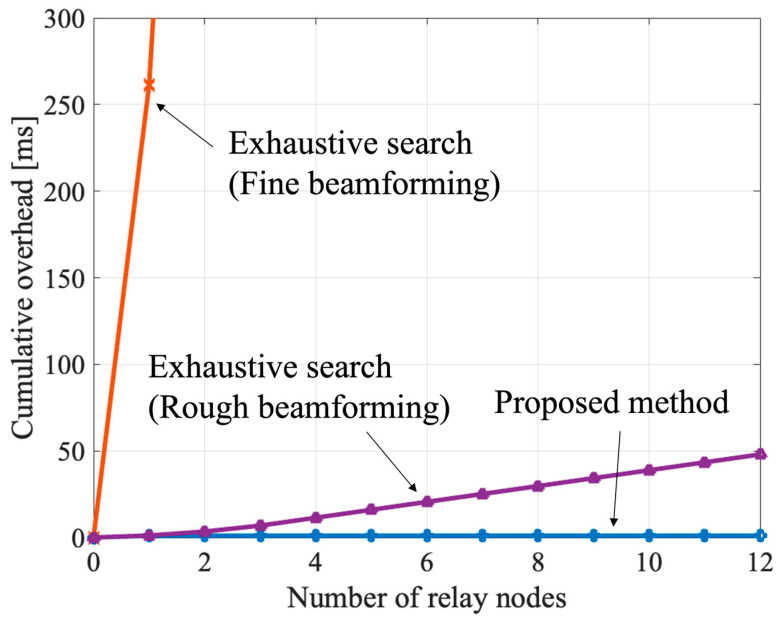
Cumulative overhead consumed to determine the relay beam on the RN access side.

We evaluated the overhead reduction by the proposed method using Equations (10) and (11). We considered a 5G NR reference signal with 120 kHz subcarrier spacing (including cyclic prefix), *T_rs_* as 17.84 μs, and CAM transmission overhead (*T_msg_*) as 1 ms. The estimated overhead is sufficient since the CAM size is less than 1000 bytes [25]. The cumulative overhead consumed to explore the relay beams is shown in Figure 8. In the exhaustive search, the overhead increases as the number of RNs. This is because the number of RNs is directly proportional to the number of beam candidates to search. In addition to that, the exhaustive search for fine beamforming is not realistic. On the other hand, the overhead of the proposed method is constant regardless of the number of RNs. Therefore, the proposed method can benefit the massive analog-relay MIMO while reducing overhead.

### 4.3. Impact of Location Error

Since the proposed method uses location information, the location error affects the rate. The location error is caused by UE positioning accuracy, control delay, and control cycle. The UE positioning accuracy depends on the location estimation method. The control delay is the time interval between the location estimation by the UE to the beam setting by the RN. The control cycle is the update cycle of the UE’s location. If the control delay and cycle is short, the update of the position information is fast, and if it is long, the update of the position information is slow. The control delay and cycle are related to UE’s mobility. If the UE is fixed, there is no location error due to the control. However, location error plays a significant role if we consider UE with high mobility.

#### 4.3.1. Impact of UE Positioning Accuracy

The impact of positioning accuracy on the proposed method is evaluated. Here, the positioning accuracy is defined as an average of the location estimation error $\left\|p_{e}\right\|$. It is assumed that the $p_{e}$ follows a normal distribution with independent *x*- and *y*-coordinates. Then, the $\|p_{e}\|=\sqrt{x_{e}^{2}+y_{e}^{2}}$ follows the Rayleigh distribution. Therefore, the relationship between the positioning accuracy α and the standard deviation σ of $p_{e}$ is given below.

(12) $$\sigma =\alpha \sqrt{\frac{2}{\pi }}$$

**Figure 9:**
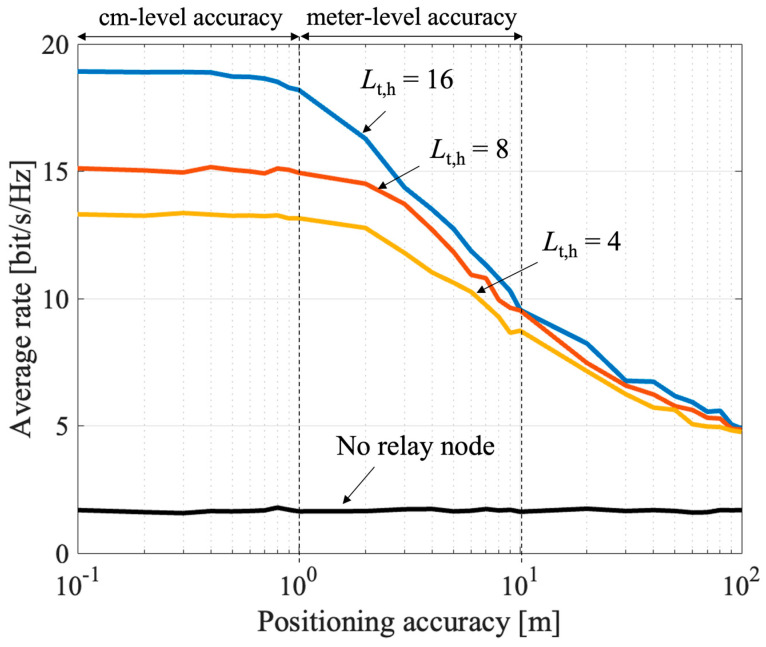
Impact of location estimation error on the UE with high-precision positioning.

The impact of the positioning accuracy is shown in Figure 9. Here, the UE location is fixed and placed in (100, 0). The number of streams is set to four. As the positioning accuracy worsens, the average rate is also reduced. However, if the centimeter (cm)-level positioning accuracy is satisfied, the rate hardly drops. LiDAR can satisfy this condition because of its cm-level accuracy. The rate tends to drop at meter-level accuracy; however relatively high rates can be maintained. Therefore, even if GNSS is applied, the effect of the proposed method can be maintained efficiently.

To mitigate the impact of positioning accuracy, another approach is to broaden the beam width. Therefore, a comparison was made with different beam widths. The beam width was adjusted by changing the number of relay antenna elements (access side) in the horizontal direction $L_{t,h}$. When the RN has a wider beam width, the impact of the positioning accuracy is mitigated, but the requirement for meter-level positioning accuracy remains. Further, as the beam width increases, the beam gain becomes small, and so the overall rate is reduced. Therefore, it is desirable to combine the proposed method with a highly accurate positioning method in order to obtain a higher rate. The results show that the proposed method works effectively by utilizing cm-level positioning methods such as LiDAR, that are expected to be available in future V2X terminals.

#### 4.3.2. Impact of Location Error Due to UE Mobility

**Figure 10:**
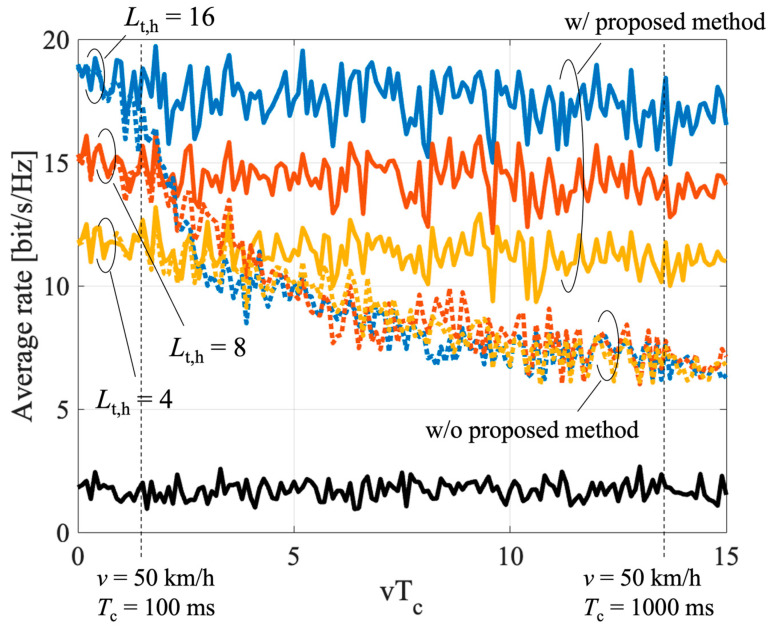
Average rate of the proposed relay-beam tracking.

The effect of the proposed relay beam tracking is evaluated in this subsection. To focus on the control of the RN, the control latency for searching the beam of the BS and the UE is ignored. In the simulation, we assume that the UE estimated its location with no error at (100, 0). Furthermore, the effect of wider beam width is also evaluated. The result is shown in Figure 10. Here, the UE speed and the beam control latency are represented by *v* and *T*_c_, respectively. Without the compensation of the mobility error, the rate decreases rapidly as *vT*_c_ increases. As the beam width of the RN narrows, the impact of the mobility error is relatively small. However, the overall rate is reduced due to the lower beam gain. The proposed method can suppress the rate of degradation without reducing the beam gain. The RN can effectively track the beam by utilizing the CAM information.

## 5. Conclusions

This paper proposes a user-driven relay beamforming method of massive analog-relay MIMO for V2X applications. The proposed method enables relay beam search and tracking in analog relays and can reduce the overhead of beam control. To reduce the overhead, the RNs utilize the UE’s location, speed, velocity, acceleration, and direction. The UE information is notified by using CAM. Simulation results show that the proposed method can benefit from massive analog-relay MIMO and also significantly reduce the overhead. One result indicates the massive analog-relay MIMO with the proposed method can improve the rate by about 72% due to the multiplexing gain of 4-streams MIMO. The accuracy of the location information, the control period, and the effects of UE mobility are also evaluated and presented. The result clarifies that it is desirable for the terminal to have cm-meter level positioning when applying the proposed method. Further, we show that the proposed relay beam tracking method works effectively with the impact of UE’s mobility and the cycle of the UE location update. We plan to extend this proposed low overhead method to a joint optimization of beam search and tracking control for a multi-user system in the future. 

## Figures and Tables

**Table 1:** Simulation parameters.

Parameter	Value
Center frequency	28 GHz
Transmit power	30 dBm @ 100 MHz
Number of BS antennas	64 (V: 8, H: 8)
Number of BS beam candidates	64 (V: 8, H: 8)
Number of UE antennas	8 (V: 1, H: 8)
Number of UE beam candidates	8 (V: 1, H: 8)
Noise power density	−174 dBm/Hz
Noise figure in UE and RN	5 dB
Number of RN antennas (Backhaul) *L*_r_	64 (V: 8, H: 8)
Number of RN antennas (Access) *L*_t_	128/64/32 (V: 8, H: 16/8/4)
Number of RN beam candidates	Fine: 14,641 (V: 121, H: 121) [24] Rough: 64 (V: 8, H: 8)
Number of RNs	12
Amplifier gain	40 dB
Pathloss model	3GPP TR 36.910 (UMi)
Array antenna type	Planar linear array
Analog beam weight	Steering vector
MIMO signal processing	SVD

## Data Availability

Not applicable.
